# Psychiatrists Report Praecox Feeling and Find It Reliable. A Cross-Cultural Comparison

**DOI:** 10.3389/fpsyt.2021.642322

**Published:** 2021-03-05

**Authors:** Marcin Moskalewicz, Piotr Kordel, Agnieszka Brejwo, Michael A. Schwartz, Tudi Gozé

**Affiliations:** ^1^Philosophy of Mental Health Unit, Department of Social Sciences and the Humanities, Poznan University of Medical Sciences, Poznan, Poland; ^2^Faculty of Philosophy, University of Warsaw, Warsaw, Poland; ^3^Texas A&M Health Science Center College of Medicine, Round Rock, TX, United States; ^4^Department of Psychiatry, Psychotherapies, Art Therapy, Toulouse University Hospital, Toulouse, France; ^5^Equipe de Recherche sur les Rationalités Philosophiques et les Savoirs (ERRaPhiS-EA 3051), Toulouse University–Jean Jaurès, Toulouse, France

**Keywords:** schizophrenia, praecox feeling, diagnosis, psychopathology, phenomenology, bizarreness, expertise

## Abstract

**Background:** The psychopathological notion of the Praecox Feeling (PF) refers to an experience of strangeness and bizarreness that arises in a clinician during contact with a patient with schizophrenia. There is evidence that psychiatrists take advantage of this feeling in their diagnostic decisions despite the domination of an operationalized diagnostic approach.

**Methods:** The article presents the results of a survey assessing the self-reported prevalence of the PF among psychiatrists in Poland and compares them with data from West Germany (1962), USA (1989), and France (2017) based on the same survey.

**Results:** The study finds a consistent prevalence of reported feelings suggestive of the diagnosis of schizophrenia among psychiatrists of different cultural backgrounds and times. These feelings are independent of variables such as attitude toward schizophrenia, professional orientation, and professional experience and are considered reliable, even if not the most reliable, by the psychiatrists who have them. The study also finds that intersubjective phenomena, such as problematic affective attunement, gestures, and body language, are considered core to these feelings by the psychiatrists.

**Conclusions:** The evidence confirms that psychiatrists' feelings about patients with schizophrenia are considered diagnostically relevant and calls for more deeply investigating the nature and diagnostic significance of these feelings. The article concludes with some speculations regarding the possible benefits of recognizing the PF in facilitating a psychotherapeutic encounter with psychotic patients.

## Introduction

This article presents the results of a study investigating the prevalence of self-reports of the Praecox Feeling (PF) in diagnostic decision making among a population of psychiatrists in Poland. It compares these results cross-culturally (Germany, USA, France) and cross-historically (over 50 years). Finally, it discusses the possible implications of the findings for the structure of medical judgment in diagnosis and the potential implications of PF for the modeling of psychotherapies for psychosis.

Praecox Feeling (PF) is a highly ambiguous notion in the conceptual history of schizophrenia. It was first described by Rümke as an experience arising in the physician in contact with a person with schizophrenia. Rümke stated that it is remarkable that “it is rare for a clinician to be able to say exactly how he arrives at a diagnosis of schizophrenia” (1), the positive and negative symptoms being, in his view, non-specific. Instead, there is a specific atmosphere or hue of symptoms—an inability to come into contact with the other's personality as a whole—that induces an intuition that leads to diagnostic certainty (2). Elsewhere, Rümke describes PF as an unease in the relationship or foreclosure of empathy. PF has been considered an important determinant of medical decision making, with high diagnostic specificity referring implicitly to a specific gestalt of the schizophrenic experience (3). Other authors have argued that PF is part of an implicit perceptual process, also called “Typification” (4, 5), which allows us to grasp the singular mode of the self/world relationship. The theoretical issues of PF have been described elsewhere (6–8).

With the rise of operational diagnostic systems (ICD and DSM) and accusations of legitimizing arbitrary psychiatry, PF has fallen into disuse since the 1980s. This experiential dimension of the clinical approach has disappeared from medical teaching programs. It reappeared in scientific discussions in the last decade in the course of reflections on the validity of the clinical diagnostic approach to the spectrum of schizophrenia (9). In the absence of consistent biomarkers, the question of finding an adequate phenomenological marker for a rapid and precise diagnosis has arisen (10). Some scholars have argued that PF is a phenomenological marker of specific alteration of basic self-consciousness (11) and intersubjectivity (12–14). And since the basic sense of self may be a core phenotypic marker of schizophrenia spectrum disorders, it has been shown that acknowledging PF in a clinical examination can predict a deteriorating course of illness in populations with a high risk of psychosis (15, 16).

Very few empirical studies have produced evidence of PF's validity and reliability compared to the operational approach. Grube's study of 67 patients with acute positive psychotic symptoms measured the intensity of PF in an experienced clinician during a few minutes long interview. This intensity was compared to standardized diagnostic classifications assessed independently. The validity of PF was high (Sensibility = 0.88; Specificity = 0.82) (17). Ungvary et al. study of 102 patients (37 with schizophrenia) assessed the intensity of PF among five psychiatrists, which was then compared to the results of a Structured Clinical Interview for DSM-IV. This study showed very inconsistent results between the five evaluators and showed poor sensitivity and specificity (18).

Regarding the prevalence of PF use in clinical practice, a study based on a self-assessed questionnaire in Germany in the pre-DSM era (19) showed a significant prevalence of psychiatrists reporting their experiences as a reliable indicator of diagnosis (85.9%). A study was carried out with the same questionnaire in the USA in the DSM-III era (20) with a similarly high prevalence (82.9%). It was replicated recently in France in the DSM-5 era (21) with comparable results (90.1%). This suggests that psychiatrists continue to take advantage of their feelings for diagnostic decisions despite the apparent contradiction of this phenomenon with operational and criteriological methods.

## Methods

### The Questionnaire

We used a simplified version of Irle's original questionnaire from 1962 from West Germany (19); it was adapted by Sagi and Schwartz (20) in a survey conducted in New York, and re-adapted by us for a study in France in 2017 (9). We retained the key questions unchanged for cross-cultural and cross-historical comparison.

The key questions of interest concerned: (1) attitudes toward schizophrenia (options: essentially incurable, only improvable with remaining deficiencies, occasionally fully reversible); (2) the possibility of a rapid diagnosis by a skilled psychiatrist (yes/no); (3) self-reported feelings strongly suggestive of the diagnosis of schizophrenia (yes/no), which, following previous interpretations by Irle, Sagi, and Schwartz, and ourselves, were considered an indicator of PF. Those having the feelings in questions were asked to fill out part 2 of the survey concerning (4) the perceived reliability of these feelings (options: usually reliable, often wrong about them); and in case of usual reliability a further qualification (5) whether, despite occasional mistakes, they are more reliable than all other symptoms (options: yes/no); (6) whether they are expressible in words (yes/no); (7) whether they consider them a result of professional experience or an experience of “strangeness” that a layman would have as well. Finally, (8) we added an extra multiple-choice question on their perceived origin, with possible answers going back to Rümke's initial speculations as well as other possibilities (options: delusional or hallucinatory experience, problematic social cognition, problematic affective attunement, gestures and body language, gaze, other—please indicate).

### Sampling and Respondents

The web-based multi-centric survey purposively targeted psychiatrists from major university clinics and psychiatric hospitals across Poland in late 2019/2020, located in Bialystok, Branice, Bydgoszcz, Cracow, Drewnica, Gdansk, Gniezno, Katowice, Koscian, Koszalin, Lodz, Lublin, Lubliniec, Miedzyrzecz, Olsztyn, Poznan, Plock, Pruszkow, Radom, Rybnik, Stronie Slaskie, Swiecie, Slupsk, Szczecin, Torun, Warsaw, Wroclaw. This was based on the assumption that clinicians working in major psychiatric clinics and hospitals more regularly deal with patients with schizophrenia. After reaching little <200 responses, the survey was circulated on a few psychiatrists' mailing lists. In order to avoid any negative or positive bias in psychiatrists familiar with the concept of PF, the invitation letter mentioned neither PF nor feelings suggestive of the diagnosis of schizophrenia—it merely asked for filling out a survey concerning the diagnosis of schizophrenia.

Our Polish survey was filled out by 243 psychiatrists, 152 female and 91 male, both specialists and residents in training, with self-reported professional orientation varying from biological, through bio-psycho-social, to psychodynamic (see Table 1).

**Table 1:** Sample characteristics.

Sample size	*N* = 243
Sex	62.6% female (*N* = 152)
	37.4% male (*N* = 91)
Expertise level	51.4% residents in training (*N* = 125)
	48.6% specialists (*N* = 118)
Professional orientation	51.9% bio-psycho-social (*N* = 126)
	31.6% biological (*N* = 77)
	9.5% psychodynamic (*N* = 23)
	7% other (e.g., environmental, social, spiritual, systemic, mixed) (*N* = 17)
Attitude toward schizophrenia	49.8% Improvable with remaining deficiencies (*N* = 121)
	39.1% Occasionally reversible (*N* = 95)
	11.1% Essentially incurable (*N* = 27)

## Results

### Praecox Feeling Among Polish Psychiatrists

For statistical analysis, SPSS v.22 was employed. When assessing the interdependence of the variables, we used Pearson's χ^2^ test to measure whether the differences within the Polish samples and between the international samples are statistically relevant. Since variables were only nominal, we used Cramér's *V* (φ_c_) test to measure the strength of associations between them.

As many as 89.3% (*n* = 217) of the sample of Polish psychiatrists occasionally experience feelings about a patient strongly suggestive of the diagnosis of schizophrenia. This is despite the fact that only 36.6% (*N* = 63) of the sample believe a skilled psychiatrist can diagnose schizophrenia very rapidly (i.e., within minutes of meeting the patient or even sooner). Furthermore, 77.4% (*n* = 161) of those having PF (*n* = 217) find it usually reliable. Females find it reliable more often (84.7%) than males (66.7%, *p* < 0.05), but the relationship with gender is not that strong (ϕ_c_ = 0.211). Only one-fourth of those who have PF (24.9%, *n* = 54) believe it is more reliable than all other symptoms (see Table 2).

**Table 2:** Praecox feeling in Poland (*N* = 243).

Rapid diagnosis possible	36.6% (*N* = 63)
PF as present	89.3% (*N* = 217)
PF as expressible in words	44.9% (*N* = 109)
PF as reliable	66.3% (*N* = 161)
PF as most reliable	22.2% (*N* = 54)

The sample disclosed different attitudes toward schizophrenia, which is important given that historically PF advocates were rather Kraepelinian and believed that schizophrenia is degenerative (22, 23). Here, only 11.1% expressed the view that schizophrenia is essentially incurable, with 39.1% opting for occasionally reversible, and 49.8% for only improvable with remaining deficiencies. Interestingly however, these attitudes do not correlate with psychiatrists having or not having PF.

Also, psychiatrists' professional orientation (51.9% bio-psycho-social, 31.6% biological, 9.5% psychodynamic, 7% other) does not correlate with any of the abovementioned PF measures, except for the ability to express it in words. In the case of biological psychiatrists, it is significantly lower (36.8%) than in all the others (57.5%, *p* = 0.005, φ_c_ = 0.193).

Finally, psychiatrists report that the feelings in questions are due to problematic affective attunement (83.6%, *n* = 173), gestures and body language (58%, *n* = 120), problematic social cognition (56.5%, *n* = 117), and gaze (55.1%, *n* = 114), while only (38.6%, *n* = 80) ascribe it to delusional or hallucinatory experience (a multiple-choice question so values do not sum up to 100%). These answers correspond to Rümke's original exposition of the source of PF.

### Cross-Cultural Comparison: West Germany, New York, France, and Poland

Our 2017 French study based on the same survey indicated that 90.1% of the sample declared feelings strongly suggestive of the diagnosis of schizophrenia—a result significantly higher than in previous West Germany (1962) and New York (1989) studies (*p* = 0.015). Seventy-four percent found it reliable—a significantly larger proportion (*p* = 0.009) than in previous studies, and 12.3% declared it more reliable than all other symptoms—a significantly lower proportion (*p* = 0.004) than in previous studies (21). Therefore, we speculated earlier about a possible French exception regarding the presence of PF and its reliability. However, the Polish study results are very close to the French one in terms of PF presence (89.3%) and to West Germany and New York ones in terms of PF being considered more reliable than all other symptoms (22.2%). The comparison of all four studies (see Table 3) shows significant differences regarding PF presence (*p* < 0.02), reliability (*p* < 0.001), and being the most reliable (*p* < 0.001), but Cramér's *V* (measuring the strength of associations between these variables) is very weak (*V* = 0.068, *V* = 0.17, *V* = 0.123, respectively).

**Table 3:** Praecox feeling across the countries.

	**Sample**				**Total**
	**West Germany**	**New York**	**France**	**Poland**	
	**1962**	**1989**	**2017**	**2020**	
PF as present^*^	85.9% (*N* = 1,027)	82.9% (*N* = 213)	90.1% (*N* = 417)	89.3% (*N* = 217)	86.8% (*N* = 1,874)
PF as reliable^**^	53.9% (*N* = 645)	64.6% (*N* = 166)	74.1% (*N* = 343)	66.3% (*N* = 161)	60.9% (*N* = 1,315)
PF as most reliable^***^	25.1% (*N* = 300)	20.7% (*N* = 53)	12.3% (*N* = 57)	22.2% (*N* = 54)	21.5% (*N* = 464)
Sample size	*N* = 1,196	*N* = 257	*N* = 463	*N* = 243	*N* = 2,159

* p < 0.02; V = 0.068;

** p < 0.001; V = 0.17;

*** *p < 0.001; V = 0.123*.

**Figure 1:**
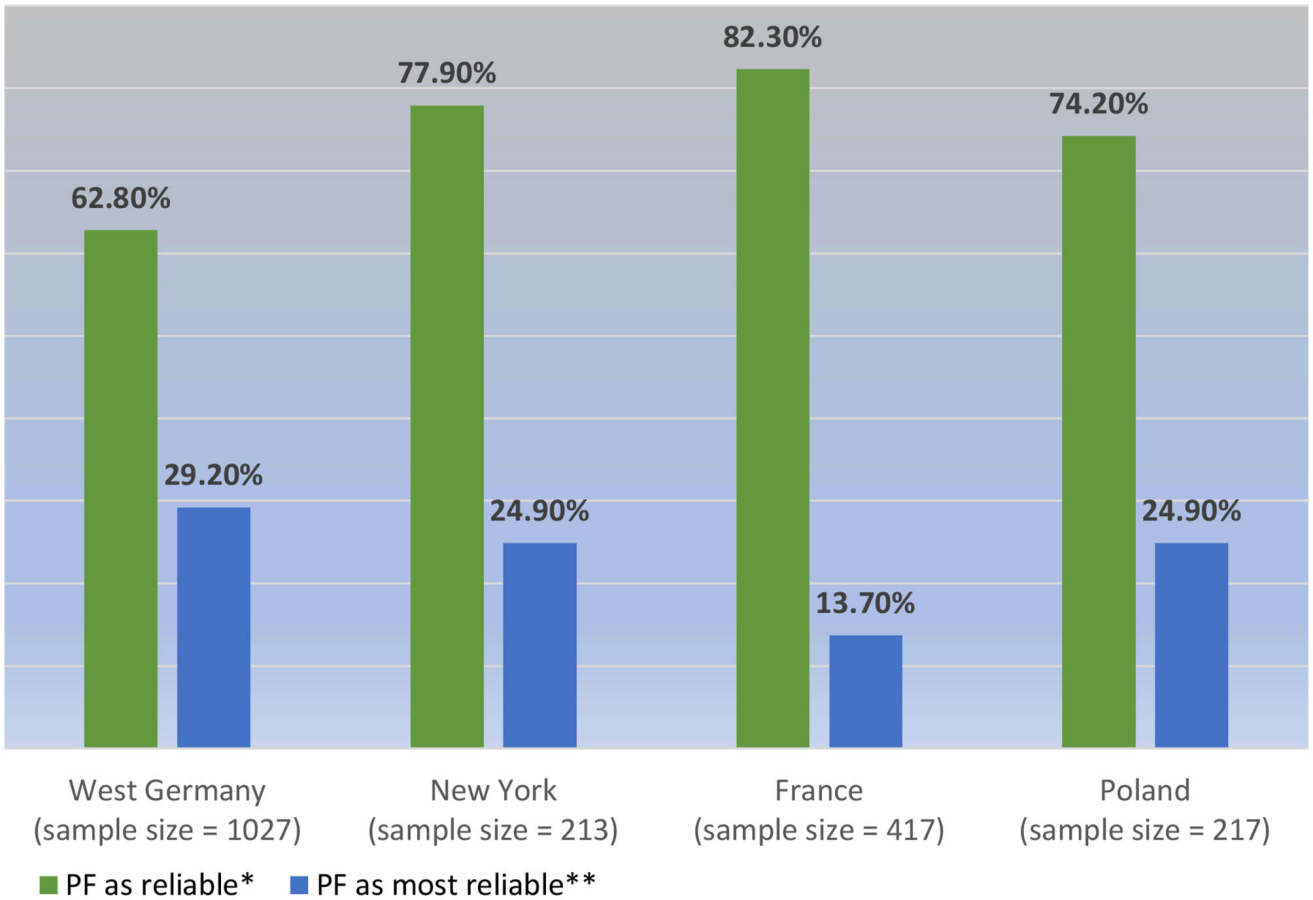
PF considered reliability in a cross-cultural sample of psychiatrists who report it (*N* = 1,874). *p < 0.001; *V* = 0.184; **p < 0.001; *V* = 0.143.

We may also combine those who report to experience PF in all four countries to construct a new artificial sample (*n* = 1874). With such a cross-cultural and cross-historical look, we find that 70.2% of all psychiatrists responding to the survey over the years considered the feelings in question reliable (*n* = 1315), but only 24.8% (*n* = 464) declared them more reliable than all other symptoms (see Figure 1). Given the low Cramér's *V*, the differences between the countries are equally negligible here.

In this light, we may conclude that there is a stable prevalence of feelings suggestive of the diagnosis of schizophrenia among psychiatrists of different cultural backgrounds and times, that the differences are negligible, and that the hypothesis of the French exception regarding PF presence (21) is unwarranted.

## Discussion

Our study indicates that PF is reported to be present in diagnostic decision-making and that is it considered reliable but not the most reliable indication by the psychiatrists themselves. For this reason, after a more thorough and experimental investigation, it could possibly guide diagnosis together with an operationalized approach. PF is also relatively independent of other variables and stable across the countries surveyed. The study also finds that what we interpret as intersubjective phenomena, such as problematic affective attunement, gestures, and body language, are considered at the core of PF by the psychiatrists who have it.

### Limitations

However, there are some limitations to these findings. For reasons explained above, the questionnaire's key question concerned feelings about a patient strongly suggestive of the diagnosis of schizophrenia and not PF explicitly. It is also important to underline that the considered reliability of PF is merely declarative, and it does not take into account the phenomenology of a singular encounter, in which it could be experimentally verified. In addition, even if the cross-cultural and cross-historical comparison is based on the same questionnaire, the studies compared were based on different types and sizes of samples. The German and the French studies used non-probability samples of the whole population of psychiatrists in West Germany and Marseille and Toulouse as well as residents from across France, respectively, but with high response rates of 51 and 25.6%. The New York study was a probability sample representative of New York County. In contrast, the Polish sample—which equaled the New York sample in size—was non-probability and mixed, and mainly targeted psychiatrists from major university clinics and psychiatric hospitals. In consequence, the international comparison conclusions cannot be representative of the psychiatrists' population.

### PF and Expertise

We have speculated earlier following the New York study findings about the possible impact of expertise on PF, namely that the number of years spent in clinical practice would affect the belief in the rapid diagnosis and its reliability (7). In the Polish sample, however, the cut-off of 25 years of professional experience considered in the New York study does not bring any statistically significant differences regarding PF's presence, its perceived reliability, or any other tested variables. The only differences appear in the cut-off between residents in psychiatry and specialists. Specialists more often believe that the presence of PF is a result of their experience as psychiatrists and not just as an experience of strangeness that a layperson could have as well (80% of specialists against 64.3% of residents; *p* < 0.05, φ_c_ 0.175); and within the total sample of Polish survey respondents with PF (both specialists and residents, *n* = 217), 71.8% believe that this is due to their professional experience. At the same time, 84.2% of specialists against 71% of residents find PF reliable *(p* < 0.05, φ_c_ = 0.157), but given the low Cramér's *V*, the association is negligible. In conclusion, based on the new data from Poland, we cannot sustain the argument that professional expertise significantly affects PF. This implies that any psychiatrist—specialist or resident—could take advantage of their feeling suggestive of schizophrenia.

### Intersubjective Alterations in Schizophrenia

We may speculate that PF refers to a rupture in dynamic interactions between two embodied self-consciousness, physician's and patient's, which produces uncertainty of their dialogical synchrony. This break is noticed through the unease of the clinician, who finds the patient un-understandable, or bizarre, as Rümke already claimed (8). Disturbances in the interactive, phenomenal field are thus crucial. We hypothesized elsewhere, following Zahavi (24) among others, that PF presumably originates in the break of primordial connection between oneself, the other, and the world (7). This idea is close to that of Minkowski, who developed the so-called penetration diagnosis (25). This concept refers to the clinician's experience that the patient has “lost vital contact with reality.” This core determinant of the psychopathology of schizophrenia is based on Bergson's vitalist philosophy and refers to the “lived synchronism” of the person and the surrounding space. We may rethink this notion as the dynamic and embodied relationship of subjectivity with the outside world, including the other. Two currents nowadays support this line of thinking: on the one hand, phenomenologically inspired psychiatrists and philosophers, and, on the other, neuroscientific proponents of 4E cognition (Embodied, Embedded, Extended, and Enacted) (26) both at times overlapping to an extent. Both argue that the human mind is not just embodied but dialogical and that experience is relational in nature (27). Consequently, mental illness is not just in the brain; it is a complex, contextual and relational process (27–29). For the remainder of this paper, we shall briefly discuss the diagnostic and therapeutic relevance of this assumption.

### Diagnostic Decision-Making

Such conceptualization backed by the Polish data favors the idea that PF is a visible side of an alteration of intersubjectivity that every trained clinician could feel. To better understand how diagnostic decision-making is structured, it is useful to distinguish two PF levels. First, there is an atmospheric change and a feeling of strangeness of the encounter, difficult to describe because it is pre-reflective and non-declarative in nature, but one that a layman can sense. Referring to Gozé (8), we call this implicit level “bizarreness of contact.” And second, this experience can be refined by the professional training of a clinician who can cognitively recognize PF as a clinical sign and refer it to his/her clinical judgment. The reflexive level refers to what Rümke called PF—a phenomenon constructed in the context of diagnostic, prognostic, and therapeutic decisions. In the diagnostic process, the tacit (pre-reflective) and explicit (reflective) PF components work in a dialectical fashion and enrich each other. Thus, PF does not stand in opposition to evidence-based criteriological diagnosis (30). Indeed, if a clinician directs the medical interview in one direction rather than another, it is based, among other factors, on where PF directs them. This is why clinicians do not explore verbal acoustic hallucinations or delusions in all patients seen in consultation; PF directs them in one direction, sometimes without thinking about it. We believe that clinicians may learn to become attentive to and skillfully explore any breaks of intersubjectivity (28), and our study calls for integrating the ability to identify one's feelings into medical training. These phenomena may likely affect structured clinical interviews, and being alert to them may increase diagnostic accuracy.

### Potential Therapeutic Significance

The results of our cross-cultural and cross-historical comparison also call for experimental investigation of the therapeutic potential of PF. Would a greater openness of the clinician/therapist to PF-like experience and the ability to identify feelings and explore phenomenal intersubjective filed significantly affect the therapeutic process?

We may speculate that being attentive to PF could facilitate psychotherapeutic encounters with psychotic patients. PF could not be just a pre-reflective sign that facilitates more reflective diagnosis, but additionally an indicator of what should be dealt with during therapy in the first place. The self-reported presence of feelings that are strongly suggestive of schizophrenia may indicate that psychiatrists are capable of detecting subtle changes in affective attunement (83.6% of the Polish sample indicate problematic affective attunement as the supposed source of their feelings).

The aforementioned break in the phenomenal field and unease of the clinician, who finds the patient un-understandable, the feeling of isolation from the Other may be a resource for realizing the need for a response that would rebuild intersubjective relatedness. In the phenomenological and hermeneutic tradition of psychopathology, what should be explored is the phenomenal field of the encounter (28). Therapy should be experiential and embodied, immersed in the present, and focused on the process rather than on the content or the past (27, 28).

One possible example of a therapeutic approach relying on an embodied and relational understanding of the mind is the Open Dialogue—a systemic and dialogical method inspired by phenomenology (27, 28). From its perspective, the psychotic experience is largely an affective response to traumatic stress. Isolation and bizarre behavior result from the lack of an adequate response (27). Open Dialogue is a consistent implementation of humanistic and phenomenological ideas that may bring about better outcomes (27, 31, 32), although more research and evidence on efficacy is needed (33). Principles such as establishing a relationship with the Other without the intention to change the Other and tolerance of uncertainty are at the heart of this approach (34). Interpersonal relatedness developed therapeutically through unconditional response to all internal and external voices in the dialogue, including non-conceptual contact, allows the creation of a language to describe the experiences manifested in symptoms (31).

## Data Availability Statement

The original contributions presented in the study are included in the article/Supplementary Material, further inquiries can be directed to the corresponding author/s.

## Author Contributions

MM, MS, and TG conceived the study and prepared the survey. AB, MM, and PK collected the data. TG wrote the introduction. MM and PK wrote the methods and results section. AB, MM, and TG wrote the discussion. All authors contributed to the article, reviewed the manuscript, and approved the submitted version.

## Conflict of Interest

The authors declare that the research was conducted in the absence of any commercial or financial relationships that could be construed as a potential conflict of interest.
